# P-83. Breaking the IV Tradition: Oral Doxycycline for MRSA Bone and Joint Infections

**DOI:** 10.1093/ofid/ofaf695.312

**Published:** 2026-01-11

**Authors:** Fernando Y Cordero Baez, Michael Martin, Jacqueline E Sherbuk, Sadaf Aslam

**Affiliations:** University of South Florida, Lakeland, FL; University of South Florida, Lakeland, FL; University of South Florida, Lakeland, FL; University of South Florida Morsani College of Medicine, Tampa, Florida

## Abstract

**Background:**

Methicillin-resistant Staphylococcus aureus (MRSA) bone and joint infections have traditionally required prolonged intravenous (IV) antibiotics. While recent trials support the non-inferiority of oral therapy, most grouped all oral agents together, limiting data on specific options like doxycycline. We aimed to assess the real-world effectiveness and safety of oral doxycycline following IV therapy for MRSA bone and joint infections.

**Figure 1:**
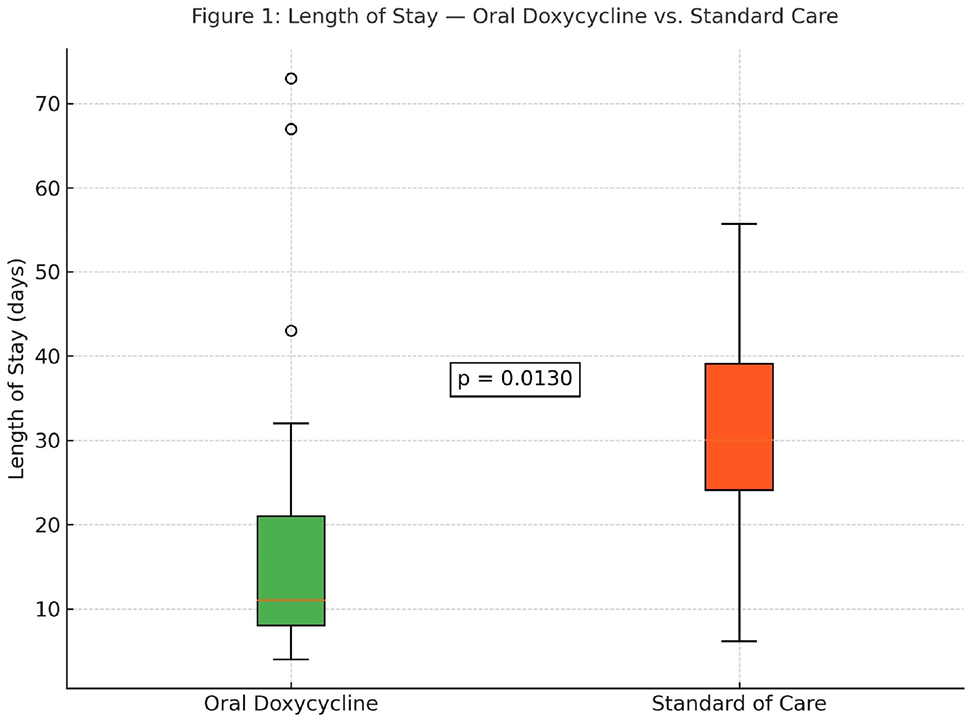
Length of Stay - Oral Doxycycline Vs. Standard of Care Comparison between our patient's mean LOS and relevant published MRSA studies.

**Table 1:**
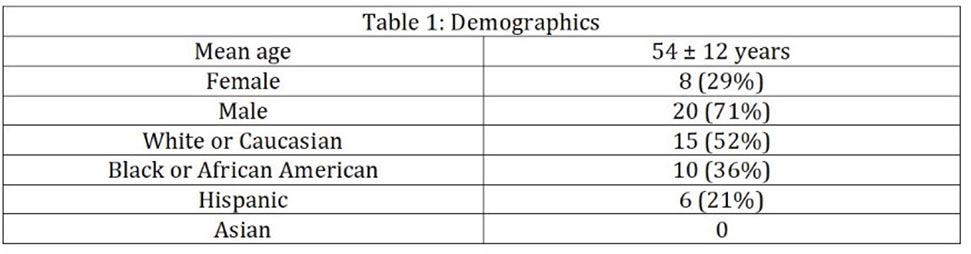
Demographics

**Methods:**

We performed a retrospective analysis of adults treated at Tampa General Hospital between January 2014 and July 2018. Patients with MRSA bone and joint infections were initially treated with IV antibiotics and later transitioned to oral doxycycline. The primary outcome was treatment success, defined as absence of culture-proven MRSA reinfection at the same site within one year. Secondary outcomes included doxycycline-related adverse events and mortality within three months. We compared the cohort’s mean length of stay (LOS) to that reported in two published IV-only MRSA studies (mean LOS ∼33 days), using Welch’s t-test on summary statistics.

**Table 2:**
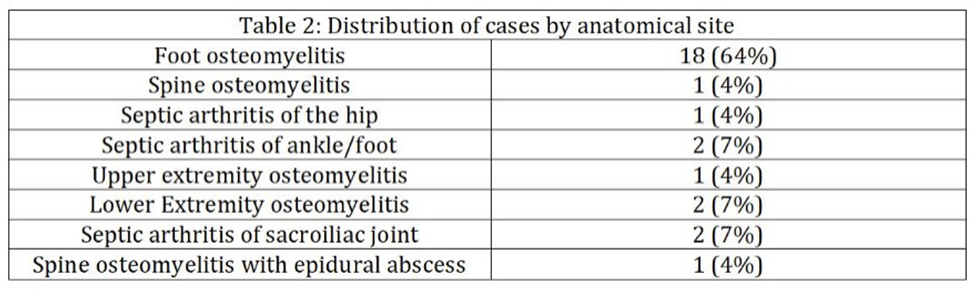
Distribution of cases by anatomical site Only culture proven infections were included.

**Table 3:**
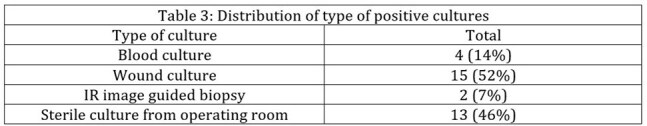
Distribution of type of positive cultures IR= Interventional Radiology. Some patients had more than one positive culture and all were included. These were the only types of cultures that were accepted as positive culture for MRSA in the study.

**Results:**

Twenty-eight patients were included. Treatment success was 96.4%, with one reinfection. No doxycycline-related adverse events or deaths occurred. Long-term suppressive doxycycline was prescribed in 10.7% of patients. Mean inpatient LOS was 18.1 days, significantly shorter than the 33-day average in IV-only cohorts (p = 0.013).

**Conclusion:**

Oral doxycycline was safe and effective in our cohort, achieving a 96.4% success rate without mortality or drug-related side effects. Compared to historical IV-only treatment, oral step-down therapy was associated with a significantly shorter LOS, potentially reducing complications and healthcare costs. An expanded cohort through 2024 is in progress to strengthen these findings and inform future treatment guidelines.

**Disclosures:**

All Authors: No reported disclosures

